# Systematic Review and Meta-Analysis of the Efficacy and Safety of Telavancin for Treatment of Infectious Disease: Are We Clearer?

**DOI:** 10.3389/fphar.2016.00330

**Published:** 2016-09-23

**Authors:** Junlan Chuan, Yuan Zhang, Xia He, Yuxuan Zhu, Lei Zhong, Dongke Yu, Hongtao Xiao

**Affiliations:** Department of Pharmacy, Sichuan Academy of Medical Sciences & Sichuan Provincial People's Hospital, School of Medicine, University of Electronic Science and Technology of ChinaChengdu, China

**Keywords:** systematic analysis, efficacy, safety, telavancin, infectious disease

## Abstract

**Objective:** Telavancin is approved to treat complicated skin and skin structure infections, hospital-acquired, and ventilator-associated bacterial pneumonia caused by *Staphylococcus aureus*. A previous meta-analysis of randomized controlled trials suggested that it might be an alternative to vancomycin in cases of difficult-to-treat meticillin-resistant *S. aureus* infections. We did a meta-analysis including one new trial to access the efficacy and safety of telavancin.

**Methods:** We searched PubMed, Cochrane Central Register of Controlled Trials, EMBASE and ClinicalTrials.gov up to December 30, 2015 to identify randomized controlled trials that assessed the clinical efficacy, eradication efficiency, adverse events and laboratory abnormalities of telavancin vs. other antibiotic agents for bacterial infection. Meta-analysis was performed using Review Manager 5.3.0.

**Results:** Five studies (3790 participants) were included in the meta-analysis. There was no significant difference in treatment success with telavancin than with control antibiotic agents. The pooled pathogen eradication for the telavancin group was numerically higher than that for the control groups, but there was no significant difference. While all-cause mortalities and serious adverse events were comparable between telavancin and control antibiotic agents, adverse event-related withdrawals (OR 1.47, 95% CI 1.13–1.91) were higher in telavancin group. The total number adverse events were more in the telavancin group than in the control groups, especially in the digestive system (OR 1.57, 95% CI 1.37–1.79), nervous system (OR 2.14, 95% CI 1.86–2.47) and urogenital system (OR 2.54, 95% CI 1.99–3.25). Serum creatinine increase (OR 2.25, 95% Cl 1.78–2.85) and hypokalemia (OR 1.74, 95% CI 1.19–2.53) occurred more frequently in telavancin group compared to control groups.

**Conclusion:** Telavancin may be as effective as but no better than the comparison therapy for *S. aureus* infection. However, because of the high risk of adverse event-related withdrawals and potential nephrotoxicity, prudence with the clinical use of telavancin in infections is required.

## Introduction

*Staphylococcus aureus* is one of the most common and virulent clinically encountered Gram-positive bacteria (Spink and Ferris, 1945). This pathogen causes serious invasive infections, such as community acquired and nosocomial pneumonia, endocarditis, soft tissue infections, and bacteremia (Drew, 2007). In 2006, results of the Surveillance Network USA showed that nearly 60% of hospital-derived *S. aureus* isolates were meticillin-resistant *S. aureus* (Styers et al., 2006). *Staphylococcus aureus* has become a major cause of hospital-acquired pneumonia (HAP) with meticillin-resistant *S. aureus* (MRSA) as the predominant pathogen. Currently, glycopeptide antibiotics such as vancomycin and teicoplanin are the gold standard for the treatment of serious infections caused by Gram-positive bacteria, especially MRSA. The emergence and prevalence of multidrug-resistant Gram-positive pathogens underscores the urgent need for development of new antimicrobials.

Telavancin is a novel lipoglycopeptide antibiotic derived from vancomycin. Telavancin exhibited concentration-dependent bactericidal effects by at least two mechanisms. It not only inhibited late-stage peptidoglycan biosynthesis in a substrate-dependent fashion, but also perturbed bacterial cell membrane potential and permeability (Higgins et al., 2005). It is intended for use to combat infections caused by *S. aureus* and other Gram-positive bacteria, including methicillin resistant and vancomycin-intermediate strains of *S. aureus* (MRSA and VISA, respectively). In the US, telavancin was approved for complicated skin and skin structure infections (cSSSI) in September 2009 and for hospital-acquired and ventilator-associated bacterial pneumonia caused by *S. aureus* in June 2013. In Europe, telavancin had been approved as second-line treatment for hospital-acquired pneumonia, including ventilator-associated pneumonia (VAP), known or suspected to be caused by MRSA when other alternatives are not suitable (Rubinstein et al., 2011a).

So far, several studies have suggested that telavancin had comparable efficacy and higher MRSA eradication rate comparing with other antibiotics for the treatment of gram-positive bacteria (Stryjewski et al., 2005, 2006, 2008, 2014; Rubinstein et al., 2011b). However, Theravance Inc., a pharmaceutical company producing telavancin (brand name as VIBATIV), was one of the affiliations of all aforementioned studies, thus creating a potential factor of biase. Some retrospective reviews and traditional reviews also studied the efficacy and safety of telavancin. However, without quality evaluation of including articles and statistical calculation of the data, the results were often influenced by subjective factors and biases (Dunbar et al., 2008; Chang et al., 2010; Hooper and Smith, 2012; Rubinstein et al., 2014; Nnedu and Pankey, 2015). Konstantinos A. Polyzos and colleagues did a meta-analysis to synthetically assess the efficacy and safety of telavancin, but it was limited to six published randomized controlled trials up to March 2012 (Polyzos et al., 2012). In addition, they only focused on the eradication of MRSA for cSSSI, but did not report the eradication of total *S. aureus* and methicillin-susceptible *S. aureus* (MSSA).

Therefore, we aimed to update Konstantinos A. Polyzos and colleagues' meta-analysis involving efficacy and safety of telavancin comparing with other antibiotics, and to give a clear insight of its clinical efficacy, adverse events and laboratory abnormalities by synthesis of the results of existing trials.

## Materials and methods

This meta-analysis was reported according to the Preferred Reporting Items for Systematic Reviews and Meta-Analyses (PRISMA) checklist.

### Data sources

A computerized search in PubMed, Cochrane Central Register of Controlled Trials and EMBASE up to December 30, 2015 was conducted independently by two individuals (Zhu and Zhong) with the search terms “telavancin” or “TD-6424.” We also did a search in ClinicalTrials.gov up to December 30, 2015 to screen completed trials about telavancin but with no published results. Furthermore, the references of retrieved literatures were manually screened for more eligible studies.

### Selection of studies

Studies that met the following criteria were considered as eligible for this meta-analysis (1) they were randomized controlled trials (RCTs); (2) studies compared the outcomes of telavancin and other antibiotic agents in infection treatment; (3) studies assessed the clinical efficacy, efficiency for eradication of pathogens, adverse events and laboratory abnormalities of both therapeutic regimens. Two independent reviewers searched the databases and screened all retrieved articles according to the inclusion criteria. Studies were excluded if they were animal studies, retrospective studies, *post-hoc* analyses, bactericidal activity studies, pharmacokinetic, or pharmacodynamic studies.

### Data extraction

Two of our authors (Zhang and He) independently extracted data for each eligible study. In cases of discrepancy, a third author (Chuan) was consulted. The following information was recorded for the included trials: study title, name of first author, year of publication, study design, type of infection, drug regimens, treatment duration, time to test of cure (TOC), number of patients, clinical and microbiological outcomes, and data on safety.

The Intention-to-Treat (ITT) population included all randomized patients in the group to which they were randomly assigned, regardless of the treatment they actually received, and regardless of subsequent withdrawl from treatment or deviation from the protocol (Fisher et al., 1989). The modified Intention-to-Treat (mITT) population referred to all patients that received at least one dose of study drug. The clinically evaluable (CE) population was patients in the mITT population who complied with all exclusion and inclusion criteria and had a clinical response of either cure or failure as assessed at the TOC visit. The microbiologically evaluable (ME) population consisted of CE patients who had a gram-positive pathogen recovered from baseline respiratory specimens or blood cultures (Stryjewski et al., 2006).

### Assessment of risk of bias

The quality of included studies was evaluated according to The Cochrane Collaboration's tool for assessing risk of bias: random sequence generation; allocation concealment; blinding of participants, personnel and outcome assessment; incomplete outcome data or selective reporting; and other sources of bias (Higgins and Green, 2011). One point was awarded for each criterion, with a maximum score of 5. Studies scored 3 or more were considered as high quality studies, whereas studies scored 2 or fewer points were classified into low-quality studies (Tasina et al., 2011).

### Outcomes

The primary outcome examined in the meta-analysis was treatment success in the mITT, CE, and ME populations, which was defined as resolution of clinically significant signs and symptoms associated with cSSSI, HAP or other infection diseases, or was defined as improvement to the extent that the infectious process had been controlled and no further antimicrobial therapy was necessary. Secondary outcomes included the pathogen eradication, adverse effects (AEs) and laboratory abnormalities. Eradication of pathogens was based on ME populations. Adverse effects and laboratory abnormalities were assessed in mITT populations.

### Statistical analysis

The statistical analyses were carried out in Review Manager (version 5.3.0) (Cochrane Collaboration, Oxford, United Kingdom). Random effects model (REM) was chosen since the included studies involving different infections, different control regimens, different sample size, which introduced obvious heterogeneity across the trials. Odds ratio (OR), with 95% confidence interval (CI), was used for all primary and secondary outcomes by Mantel-Haenszel method. The publication bias was not assessed due to the small number of the included studies. Sensitivity analysis was performed to investigate the influence on the overall results by omitting a single trial at a time.

## Results

### Selected studies and their characteristics

**Figure 1:**
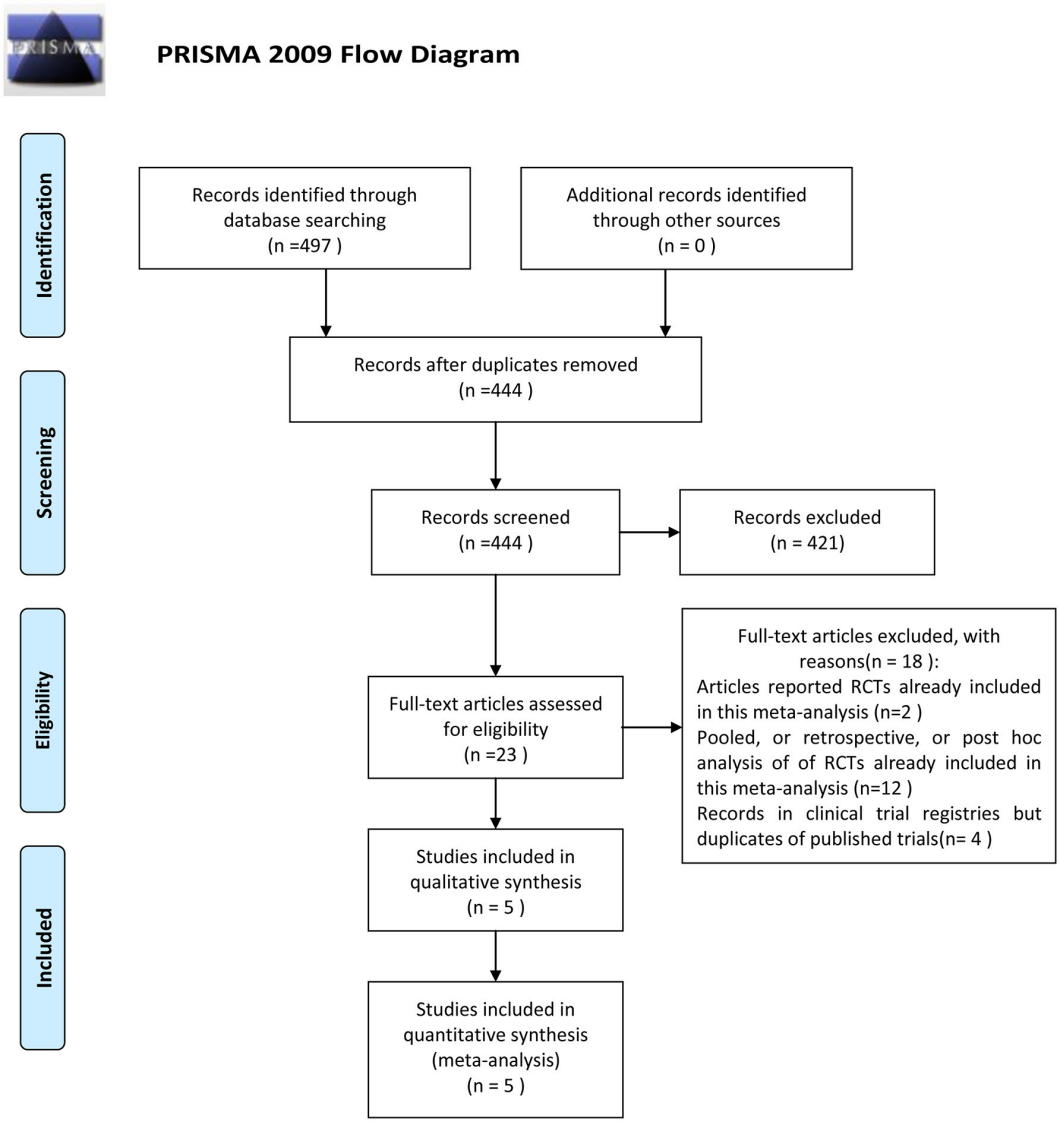
**PRISMA Flow Diagram of the Meta-analysis**.

The outcome of the search was shown in Figure 1. Four hundred and ninety seven papers were identified after initial searching. Fifty three articles were excluded because of repeated reports. Among the remaining studies, 421 studies were excluded after reading titles, abstracts or texts. Only 23 were retrieved full text for eligibility, of which 18 studies were excluded (Corey et al., 2007b,a, 2014; Stryjewski et al., 2008, 2012, 2013; Wilson et al., 2009; Barriere, 2010, 2014; Rubinstein et al., 2011a, 2014; Hooper and Smith, 2012; Nannini et al., 2012; Barriere et al., 2014a,b; Torres et al., 2014; Lacy et al., 2015). Thus, five studies comparing telavancin with control regimens were included in the meta-analysis (Stryjewski et al., 2005, 2006, 2008, 2014; Rubinstein et al., 2011b; Figure 1).

Baseline characteristics of the studies included in this analysis were presented in Table 1. All 5 studies were multicentre double-blind trials and privately funded by the pharmaceutical industry. The average age of participants was 42.3–60 years. Three studies received quality scores of 3 and the other two received 2 (Table 1). Three studies involved patients with complicated skin and skin structure infections (cSSSI) and compared telavancin intravenous (IV) at 10 mg/kg/24 h or 7.5 mg/kg/24 h with standard therapy (1 g of vancomycin every 12 h, 2 g of nafcillin or oxacillin every 6 h, or 0.5–1 g of cloxacillin every 6 h; Stryjewski et al., 2005, 2006, 2008). One study involved patients with hospital-acquired pneumonia and compared telavancin at 10 mg/kg/24 h with vancomycin at a dosage of 1 g IV every 12 h (Rubinstein et al., 2011b). One study involved patients with uncomplicated *S. aureus* bacteremia and compared telavancin IV at 10 mg/kg/24 h with standard therapy (vancomycin 1 g IV q 12 h, or nafcillin 2 g IV q 6 h, oxacillin 2 g IV q 6 h, or cloxacillin 2 g IV q 6 h; Stryjewski et al., 2014).

**Table 1:** **Main characteristics of the studies included in the meta-analysis**.

**Studies**	**Study design**	**Type of infection**	**Drug Regimen**		**Treatment duration (day)**	**Time to TOC (day)**	**Telavancin vs. comparator group (No. of patients)**			**Quality score**
			**Telavancin**	**Comparator**			**mITT**	**CE**	**ME**	
Stryjewski et al., 2014	MN,DB,Phase II RCT	SAB	10 mg/kg q 24 h	vancomycin 1 g q 12 h, or nafcillin or oxacillin or cloxacillin 2 g q 6 h	12–15	84	29 vs. 29	8 vs. 9	8 vs. 9	3
Stryjewski et al., 2006	MC,DB,Phase II RCT	cSSSI	10 mg/kg q 24 h	vancomycin 1 g q 12 h, or nafcillin or oxacillin 2g or cloxacillin at 0.5 to 1 g q 6 h	4–14	7–14	100 vs. 95	77 vs. 77	64 vs. 57	3
Stryjewski et al., 2005	MC,DB,Phase II RCT	cSSSI	7.5mg/kg q 24 h	vancomycin 1 g q 12 h, or nafcillin or oxacillin 2g or cloxacillin at 0.5 to 1 g q 6 h	4–14	7–14	84 vs. 83	72 vs. 69	56 vs. 56	3
Rubinstein et al., 2011a,b	2 MC,DB,Phase III RCTs	HAP	10 mg/kg q 24 h	vancomycin 1 g q 12 h	7–14	7–14	749 vs. 754	312 vs. 342	243 vs. 237	2
Stryjewski et al., 2008	2 MC,DB,Phase III RCTs	cSSSI	10 mg/kg q 24 h	vancomycin 1 g q 12 h	7–14	7–14	928 vs. 939	745 vs. 744	527 vs. 536	2

*Abbreviations: MN, multinational; MC, multicenter; DB, double-blinded; vs., versus; RCT, randomized controlled trial; TOC, test of cure; mITT, modified Intention-to-Treat; CE, clinically evaluable; ME, microbiologically evaluable; SAB, uncomplicated Staphylococcus aureus bacteremia; cSSSI, complicated skin and skin structure infection; HAP, hospital-acquired pneumonia*.

### Treatment success in mITT, CE, and ME populations

**Figure 2:**
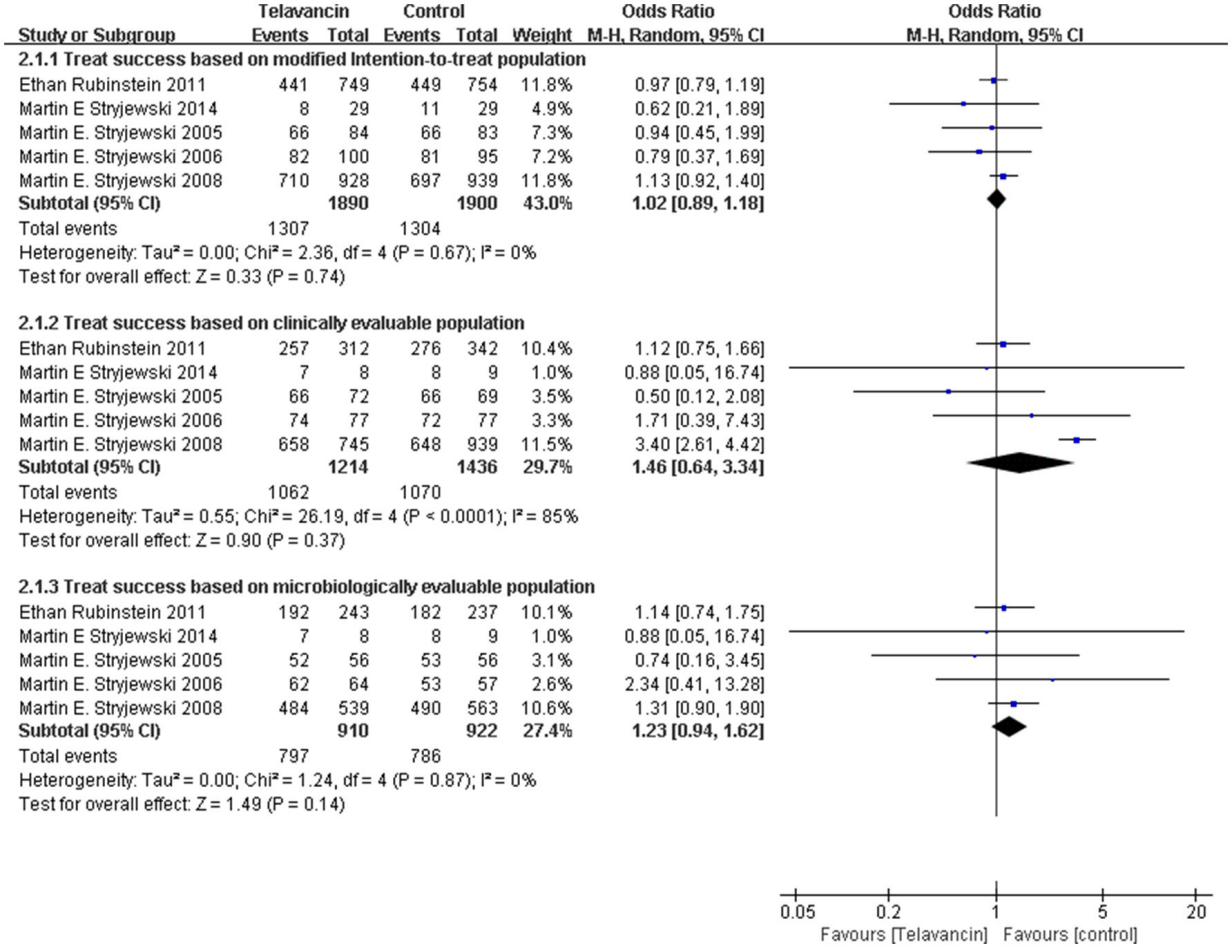
**Treatment success based on mITT, CE, and ME populations**. Df, degrees of freedom; M-H, Mantel-Haenszel method.

Assessment of treatment success was based on mITT, CE, and ME population. There was no significant difference in treatment success in the mITT population between patients treated with telavancin and those treated with comparators (5 studies, 3790 participants, OR = 1.02, 95% CI = 0.89–1.18, *P* = 0.74; Figure 2). The same was true for ME population (5 studies, 2650 participants, OR = 1.46, 95% CI = 0.64–3.34, *P* = 0.37, Figure 2) and CE patients (5 studies, 1832 participants, OR = 1.23, 95% CI = 0.94–1.62, *P* = 0.36; Figure 2). The results of two largest study groups (Stryjewski et al., 2008; Rubinstein et al., 2011b) had no effect on the overall results when they were removed one by one or both.

### Pathogen eradication in ME population

**Figure 3:**
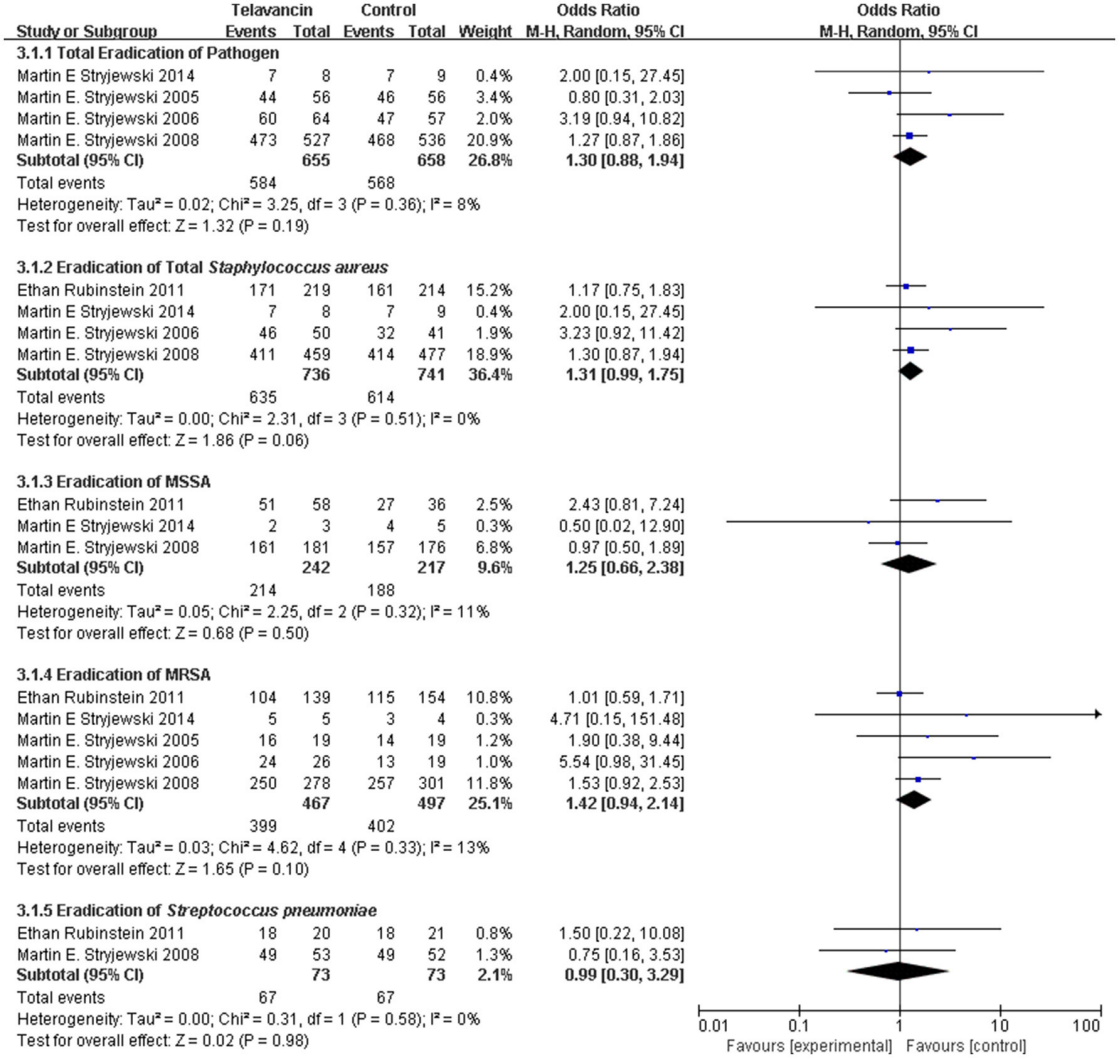
**Pathogen eradication in total and for total ***Staphylococcus aureus***, MSSA, MRSA, and ***Streptococcus pneumonia*****. Df, degrees of freedom; M-H, Mantel-Haenszel method.

The total pathogen eradication for the telavancin group was numerically higher than that for the comparator group in the ME population at the TOC visit, but there was no significant difference (4 studies, 1313 participants, OR = 1.30, 95% CI = 0.88–1.94, *P* = 0.19, Figure 3). More specifically, treatment with telavancin was associated with numerically higher eradication rate for total *S. aureus*, MSSA (for *S. aureus*, 1477 strains, OR = 1.31, 95% CI = 0.99–1.75, *P* = 0.06; for MSSA, 459 strains, OR = 1.25, 95% CI = 0.66–2.38, *P* = 0.50, Figure 3). Treatment with telavancin was associated with almost the same eradication rate for MRSA and *Streptococcus pneumoniae* (for MRSA, 964 strains, OR = 1.42, 95% CI = 0.94–2.14, *P* = 0.10; for *Streptococcus pneumoniae*, 146 strains, OR = 0.99, 95% CI = 0.30–3.29, *P* = 0.98, Figure 3). However, there were no significant differences in eradication for all these species.

### Adverse effects

**Figure 4:**
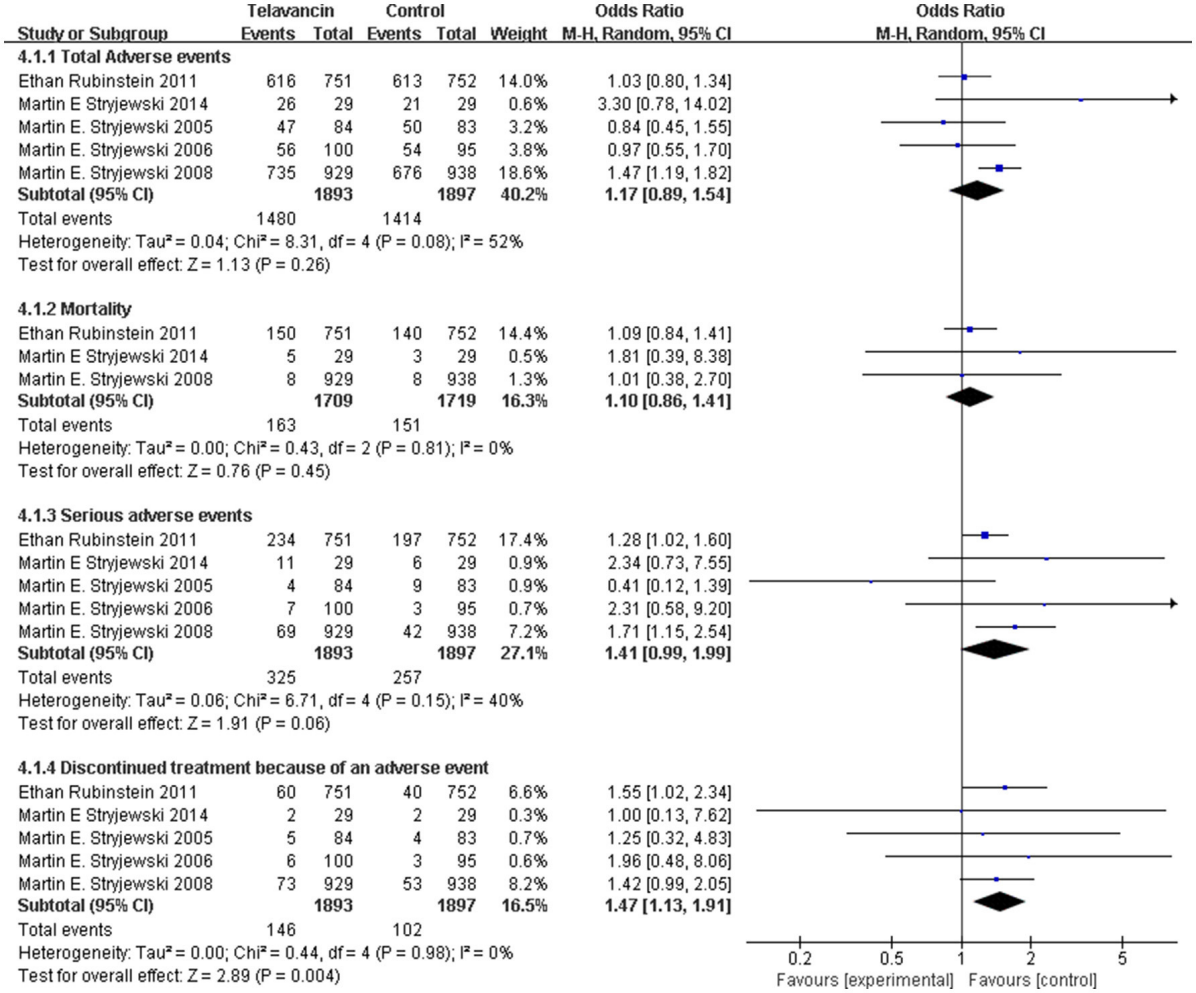
**Total adverse events, mortality, serious adverse events and withdrawal related to studied medications**. Df, degrees of freedom; M-H, Mantel-Haenszel method.

**Figure 5:**
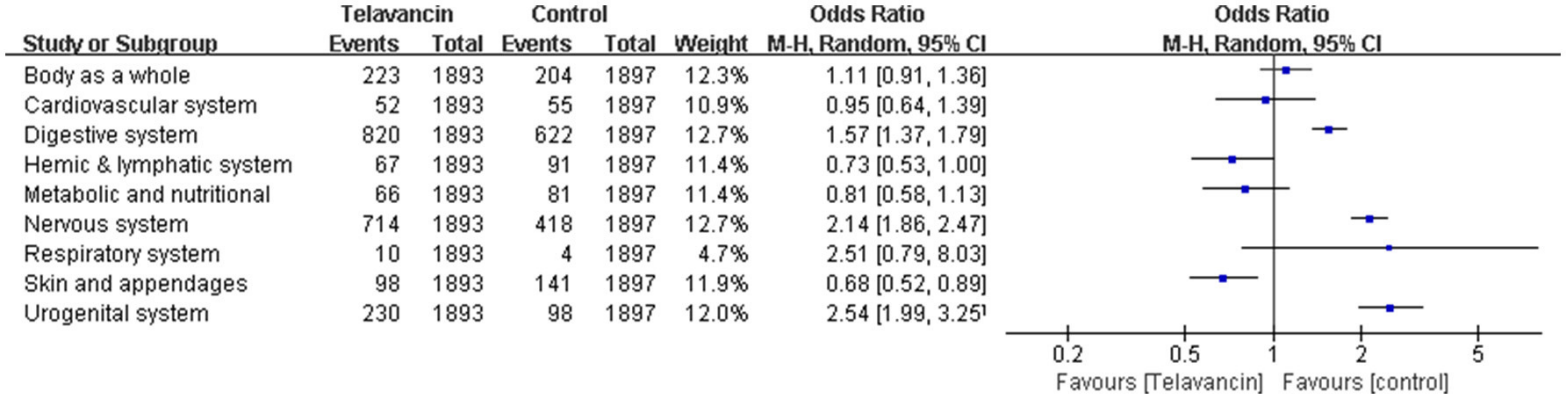
**Detailed adverse events of telavancin vs. comparator antibiotics**. Pooled odds ratios were calculated from random-effects models with the Mantel-Haenszel method.

The total number of adverse events in the telavancin groups was higher than the number in the comparators in the mITT population, but there was no significant difference (5 studies, 3790 participants, OR = 1.17, 95% CI = 0.89–1.54, *P* = 0.26; Figure 4). Overall, mortality rate (3 studies; 3428 participants, OR = 1.10, 95% CI = 0.86–1.41, *P* = 0.45; Figure 4) and serious adverse events (5 studies, 3790 participants, OR = 1.41, 95% CI = 0.99–1.99, *P* = 0.06; Figure 4) were comparable between telavancin and comparators. Discontinuance due to adverse events (5 studies, 3790 participants, OR = 1.47, 95% CI = 1.13–1.91, *P* = 0.004; Figure 4) was more common in the telavancin group based on the mITT population. Assessment of detailed adverse events showed a higher incidence in digestive system, nervous system and urogenital system in participants receiving telavancin than in control groups (Figure 5). There was no significant difference in the proportions of patients who developed adverse events in the metabolic and nutritional system, hemic and lymphatic system, cardiovascular system, respiratory system and body as a whole between the compared regimens. Significantly fewer episodes of adverse events in the skin/appendages were reported in the telavancin groups (Figure 5).

### Laboratory abnormalities

**Figure 6:**
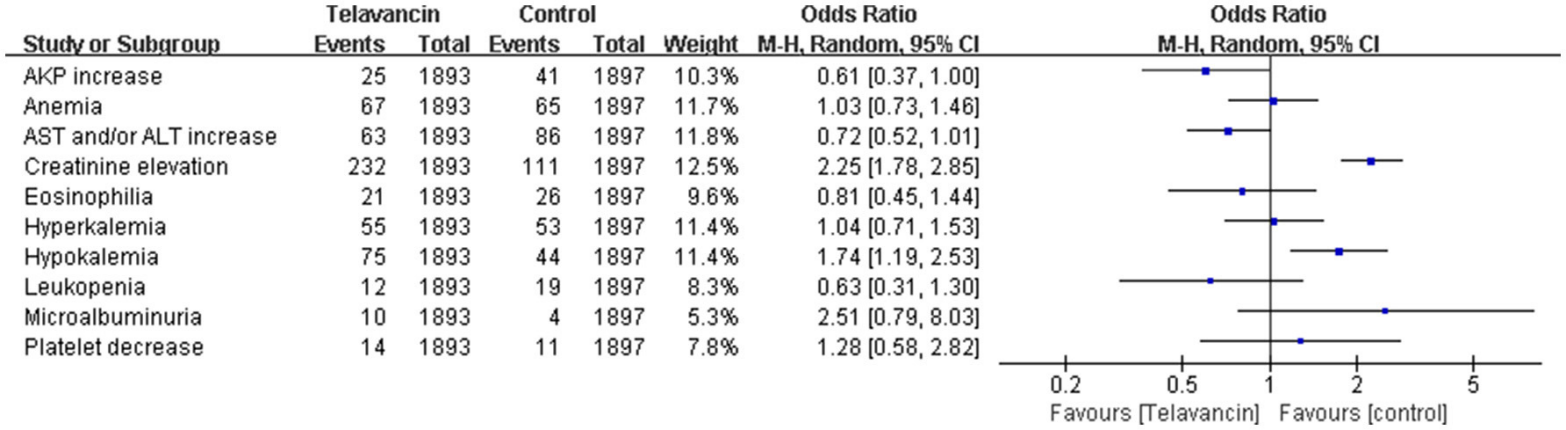
**Laboratory abnormalities of telavancin vs. comparator antibiotics**.

Significant increase in serum creatinine was more frequently observed in telavancin group compared to control group (OR = 2.25, 95% CI = 1.78–2.85; Figure 6). Moreover, hypokalemia also occurred more frequently in telavancin group than in control group (OR = 1.74, 95% CI = 1.19–2.53; Figure 6). There was no difference in alkaline phosphatase (AKP) increase, aspartate transaminase (AST) and/or alanine transaminase (ALT) increase, anemia, leukopenia, platelet decrease, eosinophilia, hyperkalemia and microalbuminuria between telavancin group and control group (Figure 6).

Pooled odds ratios were calculated from random-effects models with the Mantel-Haenszel method.

## Discussion

The present meta-analysis demonstrated the noninferiority of telavancin comparing with comparator antibiotics for cSSSI, HAP and uncomplicated *S. aureus* bacteremia. Telavancin exhibited no significant difference in eradication rate for total *S. aureus* and MRSA comparing with control group. One issue that need to be addressed was that the American Thoracic Society and Infectious Diseases Society of America (IDSA) guidelines recommended 15–20 μg/mL of vancomycin for serious infections, such as pneumonia and severe skin and soft tissue infections (Liu et al., 2011). In fact, 34% participants had a trough vancomycin level ≤ 10 μg/mL in the trials for HAP and 14% participants had a trough vancomycin level ≤ 5 μg/mL in the trials for cSSSI. The number of patients with a level ≤ 15 μg/mL was conceivably much higher. A considerable proportion of participants from vancomycin group did not receive a reasonable trough level according to the IDSA guidelines. Thus, one can only state that telavancin was not inferior to underdosed vancomycin. But it was not sufficient to claim that telavancin and vancomycin have comparable efficacy since vancomycin was underdosed (Tarchini, 2011). Therefore, telavancin is no better than standard antimicrobial regimens, for the treatment of cSSSI, HAP and uncomplicated *S. aureus* bacteremia, since it is associated with a higher frequency of withdrawl due to adverse effects, especially in the urogenital system. Among HAP patients with kidney dysfunction and pre-existing moderate-to-severe renal impairment, 28-day survival rates for telavancin were lower than vancomycin (Corey et al., 2014; Nnedu and Pankey, 2015).

In Polyzos and colleagues' meta-analysis of telavancin (Polyzos et al., 2012), results were similar to those results in our analysis for all outcomes except for eradication of MRSA. Of all 5 studies including in our mete- analysis, three studies involved patients with cSSSI, one study involving patients with HAP and one study involving patients with uncomplicated *S. aureus* bacteremia. We analyzed the synthesis eradication of MRSA for all the trials while Polyzos and colleagues only focused on that for cSSSI. In Polyzos and colleagues' meta-analysis, eradication rate of MRSA was significantly higher with telavancin than that with comparator regimens (OR = 1.71, 95% CI 1.08–2.70), whereas the difference was not significant in our study (OR = 1.42, 95% CI 0.94–2.14). The results were the same between Polyzos's meta-analysis and our study when the data from trials involving HAP and uncomplicated *S. aureus* bacteremia were removed. Thus, these results suggested that telavancin showed higher eradication rate of MRSA for cSSSI but not for HAP and uncomplicated *S. aureus* bacteremia.

There were several limitations in our study. Only one new study assessing the treatment of telavancin for uncomplicated *S. aureus* bacteremia was added in this meta-analysis. It was a multinational, double-blind phase II randomized clinical trial with 58 participants. Its sample size was too small to affect the overall synthetic outcomes. We searched the ClinicalTrials.gov for completed trials with no published data. There was only one open-label, non-randomized phase I tiral and we failed to obtain the unpublished data. Finally, all of the five included studies (7 trials) were sponsored by Theravance Inc., a pharmaceutical company producing telavancin (brand name as VIBATIV). This could be considered a factor that might introduce bias in the assessment of outcomes.

## Conclusions

In conclusion, telavancin has clinical efficacy and microbiological eradication rate similar to control antimicrobial regimens in *S. aureus* infection, including MRSA infection. However, telavancin is associated with a higher frequency of adverse events than the comparators, especially in the digestive system, nervous system and urogenital system. Because of significant serum creatinine increase and consequent potential nephrotoxicity, prudence with the clinical use of telavancin in infections is required.

## Author contributions

Conceived and designed the experiments: JC, YuaZ, and HX. Performed the experiments: JC, YuaZ, and XH. Searched the databases: YuxZ, LZ. Study inclusion: YuxZ, LZ. Data collection: JC, YuaZ, and XH. Analyzed the data: JC, YuaZ, and XH. Wrote the paper: JC, YuaZ, and HX. Critical revision of the manuscript for important intellectual content: YuaZ, DY.

### Conflict of interest statement

The authors declare that the research was conducted in the absence of any commercial or financial relationships that could be construed as a potential conflict of interest.
